# The Role of Particle Therapy in Adenoid Cystic Carcinoma and Mucosal Melanoma of the Head and Neck

**DOI:** 10.14338/IJPT-D-20-00076

**Published:** 2021-06-25

**Authors:** Daniel K. Ebner, Timothy D. Malouff, Steven J. Frank, Masashi Koto

**Affiliations:** 1 Hospital of the National Institutes of Quantum and Radiological Science and Technology (QST Hospital), Chiba, Japan; 2 Department of Radiation Oncology, Mayo Clinic, Jacksonville, FL, USA; 3 Division of Radiation Oncology, The University of Texas MD Anderson Cancer Center, Houston, TX, USA

**Keywords:** carbon-ion radiotherapy, particle radiotherapy, adenoid cystic carcinoma, mucosal melanoma, proton radiotherapy

## Abstract

Particle irradiation is suitable for resistant histologies owing to a combination of improved dose delivery with potential radiobiologic advantages in high linear energy transfer radiation. Within the head and neck, adenoid cystic carcinoma and mucosal melanoma are two such histologies, being radioresistant and lying closely proximal to critical structures. Here, we review the use of particle irradiation for adenoid cystic carcinoma and mucosal melanoma of the head and neck.

## Introduction

Particle irradiation is suitable for resistant histologies, owing to a combination of improved dose delivery with potential radiobiologic advantages in high linear energy transfer (LET) radiation. Within the head and neck (H&N), adenoid cystic carcinoma (ACC) and mucosal melanoma (MM) are 2 such histologies, radioresistant and lying closely proximal to critical structures [1]. Here, we review the use of particle irradiation for ACC and MM of the H&N.

The benefits of particle therapy are more clearly noted when compared with conventional irradiation and vary with the particle employed [2]. Three distinct particles are used today: protons, carbon-ions, and neutrons. The most common is proton, with a relative biological effect (RBE) roughly 1.1 times that of photon [3], with recent data suggesting an RBE up to 1.3 that is both cell line and fraction size dependent in H&N cell lines [4]. The beam exhibits a Bragg peak, concentrating dose within target tissue and minimizing damage to surrounding structures, with evidence of toxicity reduction vs photon [5]. At the distal end of the Bragg peak, as the LET increases, the RBE increases as well [6]. While in theory optimizing high-LET regions within the proton beam pathway could improve treatment effect [7], practically a static dose model is used.

Neutron irradiation bears a similar dose distribution to conventional radiotherapy but with notably higher LET. Consequently, increased direct nuclear damage is seen in target tissue, with an effective RBE for fast neutrons between 2 and 4 [8, 9]. Due to difficulty in collating dose, neutron therapy is uncommon today, principally used with salivary gland tumors. The carbon-ion beam arguably unites the advantages of neutron and proton, offering an RBE ∼3 with a target-focused Bragg peak [10]. Numerous centers have opened worldwide, with the first American center planned at the Mayo Clinic.

## Review Methodology

Queries of PubMed and ClinicalTrials.gov were conducted on August 5, 2020 (Table). Variation in usage of Gy, GyRBE, and Gray equivalent (GyE) was noted, and nomenclature reflects the source article.

**Table.** PubMed and ClinicalTrials.gov query results.

**Source**	**Query string**	**Results**
PubMed	“mucosal melanoma” and “carbon ion radiotherapy”	35 articles
	“mucosal melanoma” and “proton”	18 articles
	“mucosal melanoma” and “neutron”	4 articles
	“adenoid cystic carcinoma” and “carbon ion radiotherapy”	29 articles
	“adenoid cystic carcinoma” and “proton”	55 articles
	“adenoid cystic carcinoma” and “neutron”	49 articles
ClinicalTrials.gov	“mucosal melanoma”	48 studies, 1 involving proton radiation therapy
	“adenoid cystic carcinoma”	126 studies, 7 involving proton or carbon-ion radiation therapy

## Adenoid Cystic Carcinoma

Head and neck adenoid cystic carcinoma (H&NACC) is uncommon, accounting for approximately 1% of malignant H&N tumors [11]. ACC is typically characterized by an unpredictable, slow, and indolent course, exhibiting radioresistance with frequent locoregional recurrence and distant metastasis [11–13]. Standard treatment consists of surgery with adjuvant radiation therapy (RT). Given disease rarity, most data come from single institutional retrospective studies.

### Proton

Much of the data regarding proton treatment of ACC comes from Massachusetts General Hospital. A total of 23 patients with newly diagnosed ACC with skull base extension were treated with combined proton and photon therapy from 1991 to 2002. With a median of 75.9 GyE, the 5-year local control (LC) was 93% [14]. Similarly, 20 patients with primary sphenoid malignancies were reported, including 7 patients with ACC treated with a median 76.4 GyE; 2-year LC was 86% [15]. Linton et al [16] reviewed 26 patients with H&NACC treated between 2004 and 2012 with a median 72 GyE. Notably, 77% had skull base involvement. The 2-year LC was 95% for primary and 86% for recurrent disease. Four patients had grade-3+ toxicity, with 1 grade 5 [16].

Reviewing patients from MD Anderson Cancer Center, Bhattasali et al [17] presented a case series of 9 patients with unresectable H&NACC treated with definitive proton therapy and concurrent cisplatin. Four patients had a complete response at the primary site, with 4 patients experiencing stabilization of disease. One patient developed a late grade-4 optic neuropathy [17]. Moreover, 16 patients treated with postoperative intensity-modulated proton therapy to a median 60 GyE were reviewed. Twelve were given platinum-based chemotherapy, and 15 had no evidence of disease at a median 24.9 months. One patient developed a chronic grade-4 optic neuropathy [18].

Morimoto et al [19] reported on 57 patients with unresectable primary H&N cancers treated with proton or carbon-ion radiotherapy at the Hyogo Ion Beam Medical Center between 2003 and 2009. Forty-seven patients were treated with proton irradiation; most received either 65 GyE or 70.2 GyE in 26 fractions. The 3-year local progression-free (PFS) survival rate was 63% for ACC, and 3-year overall survival (OS) was 80%, although outcomes were not separated by treatment modality [19]. Additionally, 80 patients were treated with either proton or carbon-ion radiotherapy alone between 2002 and 2008, with a 5-year OS of 63% and LC of 75% [20].

Most recently, Pelak et al [21] reviewed 35 patients treated with scanning proton therapy at the Paul Scherrer Institute with H&NACC. Of the patients, 74.3% underwent surgery, while 25.7% were inoperable. Perineural invasion was noted in 71.4%. The median dose for primary and postoperative radiation treatment were 75.6 GyE and 70 GyE, respectively, in 35 fractions. The 2-year LC was 92%, with a 2-year PFS of 74%. Acute grade-3 toxicities were seen in 14% of patients, and late grade-3 toxicity was seen in 6%. No acute or late grade-4+ toxicities were reported [21].

Proton therapy has also been used with some success in case reports and small retrospective studies for treating cutaneous ACC of the cavernous sinus [22], eyelid [23], lacrimal gland [24], and nasolacrimal duct [25] with acceptable toxicity. Larger series are needed to determine the disease control rates and toxicity rates for comparison with other modalities.

Proton irradiation has been used successfully in the setting of reirradiation. Sixty-one patients with second primary tumors following previous radiotherapy were reviewed. Of the analyzed patients, 16.4% had ACC. Median dose was 66 GyE to microscopic disease, and 70.2 GyE to gross residual. The 2-year OS was 32.7% with a median OS of 16.5 months for all histologies. Grade-3+ acute toxicity was seen in 14.7% of patients and late toxicity in 24.6%, including 3 treatment-related grade-5 toxicities [26].

Massachusetts General Hospital is currently comparing intensity-modulated radiation therapy (IMRT) with proton radiotherapy in a phase II trial across H&N malignancies, including ACC (NCT01586767). Memorial Sloan Kettering has a phase II trial investigating endoscopic resection followed by adjuvant concurrent proton therapy and cisplatin for sinus and nasal tumors, including ACC (NCT03274414).

### Carbon

In 2004, the University of Heidelberg reported the early results of 21 patients treated with combination photon and carbon-ion radiotherapy (CIRT) for unfavorable ACC. Notably, the 3-year LC was 62% with no grade-3+ toxicity [27]. The first clinical results of nasopharyngeal ACC treated with primary radiotherapy were reported by Akbaba et al [28]. A total of 59 patients treated with IMRT and carbon-ion boost at the Heidelberg Ion-Beam Therapy Center were analyzed, with a 2-year LC of 83% and OS of 87%. Of the patients, 12% and 8% experienced acute and late grade-3 toxicity, respectively [28].

In Japan, Mizoe et al [29] reported a CIRT monotherapy dose escalation study of 9 patients with H&NACC between 1994 and 1997. The 5-year LC was 90%, with authors concluding that CIRT showed a “specific effectiveness” for nonsquamous histologies, including ACC [29]. In a 2012 follow-up, 236 patients with locally advanced, new or recurrent H&N cancers were treated with CIRT to 64 GyE in 16 fractions, including 69 with ACC. The 5-year LC was 73% and the OS 68%, notably higher than for other histologies [30]. In 2019, Ikawa et al [31] evaluated 43 with salivary gland carcinomas including ACC and 29 MMs treated with 56.7 to 64 GyRBE, with a 5-year LC of 78.8%; ten developed grade-3 osteoradionecrosis.

The Japan Carbon-Ion Radiation Oncology Study Group (J-CROS) conducted a retrospective review of 69 patients, including 33 patients with ACC, finding 3- and 5-year LC rates of 81% and 74%, respectively. No acute grade-4+ toxicity was seen, although 10% experienced grade-3 mucositis and 10% experienced grade-3 dermatitis. Two patients experienced grade-3 late toxicities, with one having dysphagia and another having a brain abscess [32].

Sulaiman et al [33] followed with a J-CROS review (1402 HN) of 289 histology-proven ACCs from 2003 to 2014. Approximately 69% of patients had T4 disease. After a median 64 GyRBE, at a median follow-up of 30 months, 2-year OS, PFS, and LC were 94%, 68%, and 88%, respectively. The grade 3 or higher toxicity rate was 15%, with 2 patients experiencing a grade-5 pharyngeal hemorrhage [33]. In 2018, Abe et al [34] followed with a subgroup analysis of nasopharyngeal disease, including 29 ACCs and 7 MMs treated with 64 GyRBE; 2-year LC was 88%, with 1 grade-4 optic nerve and 2 grade-5 pharyngeal hemorrhages. Ikawa et al [35] conducted a histologic analysis for solid growth pattern H&NACC, finding no difference in LC between subtypes, though there were differences in OS and PFS.

Sinonasal disease tends to be particularly aggressive and near critical organs at risk, including the optic structures. Hagiwara et al [36] analyzed 22 patients with primary sphenoid carcinoma treated with CIRT, including 15 patients with ACC. The 5-year LC rate was 51%, although 6 patients experienced grade-4 visual impairment and 1 had grade-4 necrosis [36]. Heidelberg evaluated 227 patients with sinonasal ACC with either primary or postoperative IMRT followed by scanning CIRT boost to 18 to 24 GyE. The 3-year LC was 79% for primary tumors and 82% for resected tumors. Fewer late grade-3 toxicities were seen in the primary radiation vs postoperative cohort (6% vs 17%) [37].

CIRT has demonstrated efficacy and tolerability in other disease sites, including the larynx [38], lacrimal glands [39, 40], tongue base [41], parotid glands [42], and middle ear [43].

CIRT has further shown efficacy for reirradiation. At Heidelberg, Combs et al [44] reported on 28 patients receiving CIRT to the skull base or H&N, including 4 with recurrent ACC. The median PFS for H&N tumors was 24 months [44]. At the National Institute of Radiological Sciences (NIRS), Hayashi et al [45] reported on 48 patients, including 17 with ACC and 21 with MM, with recurrent tumors between 2007 and 2016. The 2-year LC for all histologies was 40.5%, with 2-year PFS of 29.4%. Of the patients, 37.5% developed late grade-3+ toxicity, including 1 with grade-5 central nervous system necrosis [45]. Of note, a number of these patients were irradiated a third time, with only grade-2 toxicities noted.

Recently, the Italian National Center of Oncological Hadrontherapy (CNAO) reported on 51 patients with inoperable recurrent salivary gland tumors treated with CIRT as part of the phase II CNAO S14/2012C protocol. Approximately 75% of patients had ACC. With a median follow-up of 19 months, 41% of patients had stable disease and 59% had progression at time of last follow-up, with a 2-year PFS of 52% [46].

Currently, multiple investigators are conducting clinical trials on the role of CIRT for ACC [47]. The phase II COSMIC trial (NCT01154270) is evaluating IMRT of 50 Gy with 24 GyE CIRT boost to patients with inoperable or node-positive salivary gland cancer, including ACC and those with residual disease. More recently, Heidelberg opened a prospective, single-arm phase II trial in January 2020 investigating the use of CIRT alone at 66 GyE in 22 fractions, with comparison to combination photon and CIRT per the COSMIC trial (NCT04214366). In Italy, the ETOILE trial aims to compare definitive CIRT with either photon and/or proton therapy for unresectable radioresistant tumors, including ACC, chordoma, and sarcoma (NCT02838602).

Particle therapy is also being investigated in combination with different systemic therapies. In Heidelberg, the ACCEPT trial is a phase I/II feasibility trial of combined IMRT with CIRT boost and cetuximab (NCT01192087). Apatinib is being investigated for combination with particle radiotherapy for H&N ACC at the Shanghai Proton and Heavy Ion Center. In this phase II study, patients were randomized to 56 GyRBE proton followed by 15 GyRBE CIRT boost with or without apatinib 0.5 g daily (NCT02942693).

### Neutron Therapy

Although fast neutron therapy (FNT) was a promising technique, the availability is currently limited in the United States due to challenges with funding and modern national experience. Similar to other particles, neutrons have a relatively high LET, with a maximum RBE as high as 26 when treating bone marrow [9]; the inability to collate this dose led to challenges with healthy tissue toxicity in treatment cohorts, diminishing its role in modern disease treatment, particularly as carbon-ion research advanced. Most supporting data for FNT is for salivary gland tumors, which have been treated extensively from the 1970s to 1990s in the United States and Europe. Early clinical results demonstrated excellent LC, ranging from 65% to 76% [48–52]. The 10-year report from Münster, Germany, evaluated 269 patients treated with FNT from 1985 and 1995, with a 2-year OS of 76% [53]. The same group noted a complete remission rate of 39% and 1-year recurrence-free survival of 83% for the 72 patients with salivary gland ACC treated with FNT [54]. The University of Washington has reported on 84 patients with ACC, with a 5-year local regional control of 47% [55].

Most prominently, the randomized RTOG/MRC FNT study evaluated 32 patients with inoperable, recurrent, or unresectable salivary gland tumors, randomizing between FNT and photon therapy in the United States and Scotland. The complete tumor clearance was 85% for neutrons and 33% for photons [56]. At 10 years, there was a statistically significant difference in local regional control with neutrons compared with photons (56% vs 25%), although no survival difference (15% vs 25%) [57].

The most recent retrospective cohort study was reported in 2019 by Timoshchuk et al [58]. A total of 545 patients with salivary gland malignancies treated using FNT between 1997 and 2010 were identified. ACC was present in 47% of patients. The 6- and 10-year local regional control rates were 84% and 79%, respectively. Of the patients, 3% experienced osteoradionecrosis, which the authors noted was comparable to photon treatment [58]. Previous retrospective series have reported rates of oral complications after FNT for salivary gland cancers, including posttreatment trismus in 56% of patients, acute mucositis in 88% of patients, and acute xerostomia in 89% of patients [59].

### Comparison with Photon Therapy

When taken together, particle therapy provides excellent 5-year LC of approximately 65% to 90% for ACC. For instance, Samant et al [60] reported on unresectable ACC of the H&N treated with definitive photon-based radiation and concurrent chemotherapy, with a 5-year LC of approximately 61%. Furthermore, 5-year LC/locoregional control has ranged from approximately 26% to 61% [17, 60–63], with 1 study reporting a 3-year LC of 100% of the 5 patients included [64]. Toxicity data from these studies are limited, although acute grade-3 mucositis was reported in approximately 50% to 100% of patients treated with photon therapy. This is in comparison to the approximately 10% to 15% grade 3 or higher acute toxicity rate noted in proton and carbon studies, although acute toxicity is higher in some neutron studies [56].

The comparison of these results is limited, however, by the heterogeneity in both radiation technique and use with concurrent chemotherapy. For instance, studies involving ACC are often pooled with other histologies and disease sites over a prolonged period, making analysis of only ACC with a given radiation technique implausible. Regardless, particle therapy appears to have superior results to conventional photon-based irradiation, and further randomized clinical trials are unlikely to be powered effectively due to the rarity of the disease.

## Mucosal Melanoma

Accounting for less than 1.3% of all melanomas, MMs are notably rare. Half arise in the H&N, typically in the nose, paranasal sinuses, oral cavity, pharynx, and/or larynx. Notable epidemiologic variation exists between races, with MMs encompassing 8% of all melanomas in Japanese patients vs 1% in white patients, with concentration in men and ties to tobacco usage [65]. Owing to disease rarity, the literature consists principally of case series, largely concentrated in Asia. Surgery is the mainstay of treatment, though it traditionally yields >50% recurrence [66, 67]. Though adjuvant conventional radiotherapy can improve LC, a high rate of distant metastasis remains a challenge. Focused particle radiation improves therapeutic ratio with a goal to overcome radioresistance, while high-LET irradiation has been theorized to further offer improved immunogenicity, leading to enhanced systemic response [68].

### Proton

Fuji and colleagues [60] reported on a 20-patient cohort with localized sinonasal malignant MM treated with high-dose proton irradiation between 2006 and 2012. Sixteen of these patients were treated concurrently with chemotherapy. With median follow-up of 35 months (6-77 months), The 5-year OS was 51%, with PFS of 38%. The 5-year LC was 62%, with nodal/distant failure in 7 (35%), local failure in 4 (20%), and primary regrowth in 2 (10%) patients. Notably, 3 grade-4 late toxicities were observed in the tumor-involved optic nerve, and 1 patient treated with concurrent chemotherapy experienced an acute grade-4 thrombocytopenia [69].

Zenda and colleagues [70] presented a 2011 pilot study of proton beam therapy for nonsurgical treatment of MM, enrolling patients with N0M0 disease. Dose was delivered thrice weekly for 60 GyE in 15 fractions. Fourteen patients were treated, with 3 (21%) experiencing grade-3 mucositis and 2 a unilateral decrease in visual acuity without blindness. Three-year LC was 85.7%, with PFS of 25.1% and OS of 58% [70]. A phase II study followed in 2015, evaluating 32 patients. At a median follow-up of 36.4 months, 1-year LC was 75.8%, with 3-year OS of 46.1%. Of nonsurviving patients, 93.3% died due to distant metastasis [71].

In 2014, Demizu and colleagues [72] presented a mixed retrospective analysis of proton and carbon-ion irradiation encompassing 62 patients, 33 of whom received proton therapy. With a median follow-up of 18 months, the 1- and 2-year proton OS rates were 91% and 44%, with LC of 92% and 71%, respectively. No significant difference was seen between the proton and carbon arms. Five patients experienced local recurrence, and 18 experienced distant metastasis. Three experienced grade-3+ toxicity [72].

Proton therapy has also been used with success in adjuvantly treating MM of the palatine tonsil after partial pharyngectomy with left level I-V neck dissection. This patient was free of disease 11 months after surgery and 8 months after RT [73].

In the United States, Christopherson and colleagues [74] reviewed 4 patients at the University of Florida who received combined photon and proton RT. On pooled analysis, they found inferior outcomes for patients who received definitive RT as opposed to surgery alone [74]. The Washington University School of Medicine currently has a trial investigating pembrolizumab and hypofractionated RT for MM, using IMRT and intensity-modulated proton therapy in 5 fractions of 6 Gy (NCT04318717).

### Carbon-Ion

In 2004, Mizoe et al [29] conducted a phase I/II dose escalation clinical trial of CIRT, evaluating 36 patients with locally advanced, histologically proven H&N cancer, including 5 MM. Five-year LC was 100% [29]. Yanagi and colleagues [75] expanded on this with a 2009 retrospective study of 72 MM patients treated with CIRT across 3 prospective studies. Doses ranged from 52.8 GyE to 64 GyE in 16 fractions. With a median follow-up period of 49.2 months (range, 16.8-108.5 months) and no grade-3+ late toxicity, the 5-year LC, OS, and cause-specific survival were 84.1%, 27.0%, and 39.6%, respectively. Of the patients who developed distant metastasis, 85% were free from local disease [75]. In a follow-up phase II trial, Mizoe and colleagues [30] evaluated 85 patients with MM, achieving a 5-year LC of 75% with OS of 35%. Predictive microsatellite markers have been derived from this study [76].

In a 2010 systematic meta-analysis, Ramaekers and colleagues [77] compared 74 photon, 5 CIRT, and 7 proton observational studies for H&N cancer. Five-year OS of MM was significantly higher for CIRT than conventional therapy (44% vs 25%, *P* = .007). They concluded that CIRT may be preferential in radioresistant disease, while proton appears to match the best conventional results with less frequent toxicity [77].

In 2014, Demizu et al [72] conducted a retrospective study of proton vs CIRT, in which 29 patients received CIRT. With a median follow-up of 18 months, the combined 1- and 2-year OS rates were 96% and 62%, with LC of 95% and 59%, respectively. No significant difference was seen between proton and carbon arms. Three patients experienced local recurrence, and 11 experienced distant metastasis. Two patients exhibited grade-3+ toxicity [72].

In 2016, Mohr and colleagues [78] from the University of Heidelberg evaluated combined IMRT with CIRT boost. Eighteen patients, 94% of whom had T4 disease, were treated between 2009 and 2013 with a median dose of 74 GyE. No concurrent chemotherapy was delivered, and no grade-3+ toxicity was noted. Three-year OS, PFS, and local regional control were 16.2%, 0%, and 58.3%, respectively, at a median 18 months; survival was limited due to distant metastasis [78].

Koto et al [79] conducted a subgroup analysis of J-CROS 1402 HN, evaluating 260 patients with H&N MM. Eighty-six had T3, 147 T4a, and 27 T4b disease; 9 were N1. A median 57.6 GyRBE was delivered, with 129 patients receiving concurrent dimethyl traizeno imidazole carboxamide. At a median follow-up of 22 months, 2-year OS and LC were 69.4% and 83.9%, respectively; gross tumor volume and chemotherapy were significant prognosticators. Twenty-seven developed grade-3 and 7 developed grade-4 late toxicity [79].

In 2017, Naganawa and colleagues [80] retrospectively evaluated patients with oral MM at NIRS treated with definitive CIRT. Nineteen patients were noted, encompassing T3 and T4a disease; 3 patients were N1, and all were M0. Treatment of 57.6 GyRBE was delivered in 16 fractions, and with a median follow-up of 61 months, the 5-year LC, OS, and PFS were 89.5%, 57.4%, and 51.6%, respectively. Grade-2 and grade-3 osteoradionecrosis were observed in 3 and 4 patients, respectively [80].

Takayasu and colleagues [81] at Gunma University conducted a prospective observational study of combination CIRT with dacarbazine, nimustine, and vancristine for MM. Twenty-one patients with T4a or T4b rhinosinus disease were treated with CIRT to 57.6 to 64.0 GyRBE in 16 fractions, with 2 to 3 cycles of adjuvant dacarbazine, nimustine, and vancristine. With a median follow-up of 31.2 months, the 3-year LC, OS, and PFS were 92.3%, 49.2%, and 37.0% respectively. Distant failure occurred in 52% of patients. No patients developed grade 3 or higher toxicity [81].

Koto et al [82], in collaboration with J-CROS, conducted a multi-institutional subgroup analysis of locally advanced sinonasal tumors, evaluating a total of 221 MM patients (48% of the cohort) across four CIRT institutions in Japan between 2003 and 2014. Sixty-five percent had T4 disease. With a median follow-up of 25.2 months, 2-year LC for MM was 82.5%, with 68% OS and 37.5% PFS. On pooled analysis, 17% of patients developed grade-3 and grade-4 toxicities, in which visual impairment was most common [82].

### Neutron

Liao and colleagues [83] at the University of Washington retrospectively evaluated 14 patients with primary H&N MM treated with neutron between 1990 and 2012. Five patients had T3 disease, 9 had T4, 3 had regional nodal, and 4 had distant metastasis, with 10 sinonasal, 2 lip, and 2 palate-based tumors irradiated. Five-year LC was 66% with 21% OS. Survival was limited by early distant metastasis [83]. Initial work has gone into evaluating MM as a target for boron neutron capture therapy, though most is restricted to animal models to date [84].

### Comparison to Photon Therapy

Like ACC, comparison of results with MM with particle therapy and conventional photon therapy is limited by the generally low number of patients, as well as the heterogeneity of treatment techniques and pooled cohorts of various anatomic sites. For instance, treatment with surgical resection before adjuvant radiation and the use of chemotherapy confound comparison. Studies of photon-based treatments estimate a 5-year LC of 35% to 79% and 5-year OS of 16% to 45% [74, 85–89].

A complete analysis of toxicity using photon-based techniques is limited given the heterogeneity of photon technology and toxicity reporting. Hallemeier et al [90] reported on 46 patients with MM of the H&N treated with adjuvant hypofractionated IMRT and noted an 85% grade-1 to grade-2 mucositis rate with 2% acute grade-3 toxicity. Of note, no patients had late grade 3 or higher toxicity [90]. Similarly, Sas-Korczynska et al [91] noted acute grade-3 dermatitis and mucositis in 1 of 6 patients treated with definitive IMRT. One patient had grade-2 optic nerve toxicity, with no patients experiencing grade 3 or higher toxicity [91]. These data are consistent with the toxicity rates noted with CIRT and proton-based therapy, although the data are limited by small sample sizes. Further multi-institutional analyses will be needed to compare toxicities.

**Figure 1.**
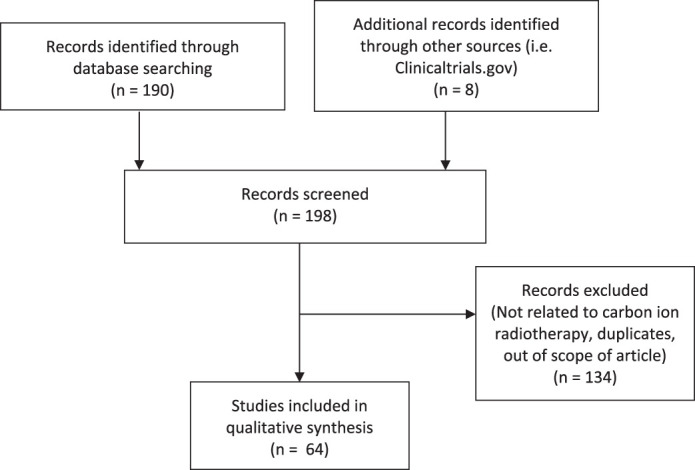
PubMed and ClinicalTrials.gov database search strategy.

## Challenges and Conclusions

ACC and MM are notably rare diseases, and the paucity of cases with geographical and demographic biases limits the generalizability of definitive treatment data. Many publications are pooled analyses, and overlap of patient cohorts between reports is likely. Similarly, patient course was noted to be heterogeneous between trials, with varying adjuvant and salvage approaches incorporated that may have gone underreported. Additionally, direct comparison between the different types of particles is impractical given the limited availability of centers treating with CIRT and neutron therapy.

Nonetheless, particle therapy appears a viable means by which to improve disease control, with the high-LET and conformal carbon-ion beam demonstrating unique promise in critical-adjacent radioresistant disease. Summarized, proton therapy and carbon-ion therapy provide excellent 5-year LC rates, ranging from 73% to 90% for ACC and 62% to 84% for MM; survival remains limited by systemic disease control. Currently, routine use of particle therapy for ACC and MM is limited primarily by the low number of centers treating with particles. Consideration for a phase III trial is complicated by disease rarity and consideration of clinical equipoise versus conventional treatment; in the absence of a prospective and randomized approach, propensity-standardized comparative exploration is warranted, and ongoing retrospective multi-institutional updates should be released as follow-up time course accrues.
